# A Bayesian mixture modeling approach for public health surveillance

**DOI:** 10.1093/biostatistics/kxy038

**Published:** 2018-09-25

**Authors:** Areti Boulieri, James E Bennett, Marta Blangiardo

**Affiliations:** Department of Epidemiology and Biostatistics, MRC- PHE Environment and Health, Imperial College London, Norfolk Place, London W2 1PG, UK

**Keywords:** Bayesian hierarchical analysis, Mixture modeling, Public health surveillance, Road traffic accidents, Small-area detection, Spatio-temporal modeling

## Abstract

Spatial monitoring of trends in health data plays an important part of public health surveillance. Most commonly, it is used to understand the etiology of a public health issue, to assess the impact of an intervention, or to provide detection of unusual behavior. In this article, we present a Bayesian mixture model for public health surveillance, which is able to provide estimates of the disease risk in space and time, and also to detect areas with unusual behavior. The model is designed to deal with a range of spatial and temporal patterns in the data, and with time series of different lengths. We carry out a simulation study to assess the performance of the model under different scenarios, and we compare it against a recently proposed Bayesian model for short time series. Finally, the proposed model is used for surveillance of road traffic accidents data in England over the years 2005–2015.

## 1. Introduction

In public health surveillance, it is typically of interest to describe spatio-temporal trends of a disease, as well as to detect areas where the disease risk follows a temporal pattern that deviates from the expected one. This can lead to important findings such as the identification of a localized risk factor, and can therefore, be a useful tool for public health researchers and practitioners. The situation considered here is the small area context, where the study region is divided into small bounded areas that typically represent administrative regions, each corresponding to an aggregated observed number of cases and an associated expected number of cases or population count.

There is a large literature on surveillance systems which can be distinguished into test statistics and model-based methods (Lawson and Kleinman, 2005; Robertson *and others*, 2010). The first class seeks to determine whether a defined subset (temporal, spatial, or spatio-temporal) is unusual compared to the incidence in the study region as a whole. Test statistics have a long history; see for instance the standard tests by Knox and Bartlett (1964) and Mantel (1967) which determine whether pairs of cases are close in space and time. These were followed by the scan statistics (Kulldorff, 1997) which normally use a circular window that passes over the study region and determines the significance of the number of cases within the window based on likelihood ratio statistics. SaTScan (Kulldorff *and others*, 2005) is perhaps the most popular scan statistic which has been extensively used for health care applications and is available through a user-friendly software (http://www.satscan.org/).

Model-based methods, on the other hand, are used to describe the underlying distribution of the data. Compared with the test statistics, they allow for the inclusion of random effects and explanatory variables to adjust the disease risk, thus providing a much more flexible framework. Bayesian hierarchical models have received considerable attention over the last two decades, due to advances in computer power allowing the implementation of sophisticated algorithms. These have been extensively applied in disease mapping studies aiming at providing estimates for the incidence of a disease (see for instance Waller *and others*, 1997; Sun *and others*, 2000; Knorr-Held and Richardson, 2003; Best *and others*, 2005; Lawson, 2013; Rushworth *and others*, 2014), while recently they have been used for the detection of change (Abellan *and others*, 2008; Zhou and Lawson, 2008; Li *and others*, 2012; Wakefield and Kim, 2013; Lee and Lawson, 2014; Napier *and others*, 2016). Li *and others* (2012) proposed BaySTDetect, a promising method specifically designed to detect areas with unusual temporal patterns for non-communicable diseases, which has been applied to several studies (Li *and others*, 2014; Duncan *and others*, 2016; Boulieri *and others*, 2016).

Briefly, BaySTDetect is a mixture of two model components: the first describes the background effect of the disease accounting for spatial and temporal correlations and estimates one time trend for the whole study region, while the second estimates a time trend for each area. A mixing parameter $z_{i}$ allocates each area $i$ to one of the two components, classifying these as common and unusual. A Bayesian estimate of the false discovery rate (FDR) is also used in order to control for the multiple testing problem. The method has shown its potential for detecting changes in time trends under various realistic scenarios, and performed better when compared to SaTScan, however, it suffers from important limitations. For instance, the mixing parameter $z_i$ is constant over time, making the method perhaps too restrictive for long time series, as it assumes that the unusual trend applies to the entire time period. In addition, the prior specification of $z_i$ does not account for spatial correlation in the unusual areas, which might produce biased results when unusual areas in the form of clusters are present.

In this article, we describe a modeling approach for epidemiological surveillance of public health data. By using BaySTDetect as the baseline model, we adopt its mixture specification and further extend it to address its limitations. Our proposed model is able to detect temporal changes in the spatial setting for scenarios where the baseline model lacks performance. The remainder of the article is structured as follows: in Section 2, we describe the baseline model, its limitations, and our proposed approach to overcome these. In Section 3, we present a simulation study to assess the performance of our model against the baseline model. A case study on road traffic accidents data in England is carried out to illustrate the method in Section 4, and we raise discussion points in Section 5.

## 2. Methods

### 2.1. Baseline model

Following the epidemiological structure on disease mapping, let $Y_{it}$ and $E_{it}$ denote disease counts and expected counts respectively in area $i=1, \ldots, N$ and time point $t=1, \ldots, T$. In the first level of the hierarchy, a Poisson lognormal model is assumed for the disease counts,

(2.1) \begin{equation*}Y_{it} \sim \mbox{Poisson}(\mu_{it} E_{it}),\end{equation*}

where $\mu_{it}$ represents the relative risk of the disease in area $i$ at time point $t$. This parameter is modelled as a mixture of two alternative models, a *Common model* and an *Area-Specific model*. The *Common model* assumes one global time trend for all areas, while the *Area-Specific model* estimates a time trend for each area independently. This aims to distinguish between areas that follow the expected time trend and those that exhibit unusual behavior. More specifically, the second level of the hierarchy models $\mu_{it}$ as follows:

(2.2) \begin{eqnarray*}\log(\mu_{it})& = & z_i \; \mu_{it}^{(C)} + (1-z_i) \; \mu_{it}^{(AS)} \text{, where}\\\end{eqnarray*}

(2.3) \begin{eqnarray*}\mu_{it}^{(C)} &=& \alpha_0 + \eta_i + \gamma_t \quad \textit{(Common model)}, \\\end{eqnarray*}

(2.4) \begin{eqnarray*}\mu_{it}^{(AS)} &=& \nu_i + \kappa_{i_t} \quad \textit{(Area-Specific model)},\end{eqnarray*}

and $z_i$ is the mixing parameter that selects between estimates $\mu_{it}^{(C)}$ from the *Common model* and estimates $\mu_{it}^{(AS)}$ from the *Area-Specific model*.

The *Common model* (Eq. 2.3) follows a standard disease mapping approach where the relative risk $\mu_{it}$ consists of a global spatial component $\eta_i$ and a global temporal component $\gamma_t$. The spatial component $\eta_i$ is assigned a convolution prior, widely known as BYM, which was proposed by Besag *and others* (1991). This is a Gaussian prior, $\eta_i \sim N(v_i,\sigma^2_\eta)$ where $v_i$ is a spatially structured term following an intrinsic conditional autoregressive prior (ICAR)

(2.5) \begin{equation*}v_i \vert \boldsymbol{v}_{-i} \sim \mbox{Normal} \left(\frac{1}{N_i} \sum_{j=1}^{N}{w_{ij}v_j}, \frac{\sigma_{v}^{2}}{N_i}\right)\!.\end{equation*}

The parameter $w_{ij}$ represents the entries of the adjacency matrix $\bf{W}$ of size $N \times N$ specifying the spatial neighborhood structure, such that $w_{ij} = 1$ if areas $i$ and $j$ share borders and $w_{ij} =0$ otherwise. $N_i$ is the number of neighbors of area $i$.

Similarly, the temporal component $\gamma_t$ follows a Gaussian random walk prior of order 1 $(\mbox{RW1})$, which is implemented as the temporal analogue of the ICAR prior in Eq. (2.5), $\gamma_t \vert \boldsymbol{\gamma_{-t}} \sim \mbox{ICAR}(\textbf{Q}, \sigma^2_\gamma)$, where the entries of the adjacency matrix $\bf{Q}$ are given by $q_{ht} = 1$ if $\vert h - t\vert = 1$ and $q_{ht} = 0$ otherwise, with $h$ and $t$ indexing units of time.

An overall intercept which follows a flat prior $\alpha_0 \sim U(-\infty, +\infty)$ is included due to the sum-to-zero constraints of the ICAR priors $\sum_{i}{v_i}=0$ and $\sum_{t}{\gamma_t}=0$ such that the model is identifiable (Thomas *and others*, 2004).

The *Area-Specific model* (Eq. 2.4) consists of an area-specific intercept that follows a weakly informative prior $\nu_i \sim N(0,1000)$ and an area-specific temporal component $\kappa_{i_t} \sim \mbox{ICAR}(\textbf{Q}, \sigma_{\kappa_i}^2)$ where the adjacency matrix $\bf{Q}$ is defined as above.

The hyperparameters $\sigma^2_\eta$, $\sigma^2_v$, and $\sigma^2_{\gamma}$ are assigned a weakly informative half Normal prior $N(0, 1)$, while $\log(\sigma_{\kappa_i}^2) \sim N(a, b^2)$, where $b \sim N(0, 2.5^2)$, bounded below by 0, and $a \sim N(0, 1000)$ (for details see Li *and others*, 2012).

The mixing parameter $z_i$ follows a Bernoulli prior assuming that around 5% of the areas are unusual:

(2.6) \begin{equation*}z_i \sim \mbox{Bern}(0.95).\end{equation*}

The model is fitted to the data set through Markov chain Monte Carlo (MCMC) sampling, and, at each iteration, the mixing parameter $z_i$ takes values 1 or 0 taking into account which model (*Common* or *Area-specific*) is more likely under the data. At the end of the MCMC sampling, the posterior mean of $z_i$, namely $f_i$ = P($z_i$ = 1$\mid$data), reflects the posterior probability that area $i$ is common or unusual over the whole time period. In order to account for the multiple testing problem, a detection rule is employed based on a Bayesian estimate of the FDR (Newton *and others*, 2004; Ventrucci *and others*, 2010). According to the rule, the areas with $f_i$ below threshold $C=f(k)$ are classified as unusual; $k$ is the maximum integer such that $1/k \sum_{j=1}^{k}{f_{(j)}} < \alpha$, with $f_{(j)}$ denoting the $j^{th}$ ordered posterior probability and $\alpha$ a preset level usually taken equal to 0.05 (for details see Li *and others*, 2012).

### 2.2. Limitations of the baseline model

The model described above has shown its potential for detection of unusual behavior within the small area context, however, it suffers from drawbacks that limit its applicability. More specifically:

1. The mixing parameter $z_i$ (Eq. 2.6) assumes that the probability that area $i$ follows the common or unusual trend remains the same across the whole time period. This assumption is perhaps too restrictive and makes the model appropriate only for short time series, (i.e. Li *and others* (2012)) tested the model on simulated data with 8 time points and recommend that it can be used for a maximum length of 10.
2. The posterior estimates of $z_i$ give information regarding the location of the unusual behavior but not the corresponding time point/points when this occurs.
3. The prior on $z_i$ (Eq. 2.6) assumes independence across unusual areas, however, in epidemiological studies these can be spatially correlated, and not accounting for this can result in unreliable estimates.
4. In addition, $z_i$ assumes that a priori 5% of areas are unusual (Eq. 2.6), which might induce bias when this does not agree with the data, for instance when no outbreaks exist or a much larger proportion of areas are unusual. This is particularly likely to happen when very sparse data are considered, i.e. short time series and/or low counts, where the prior can have a strong effect on the posterior. The impact of this prior specification on the detection power of the model has been described in Li *and others* (2011).
5. Because of the nature of $z_i$, multiple independent tests are performed and hence the multiple testing problem arises. The Bayesian FDR specification is incorporated in the baseline model to address this, however, as can be seen in the original paper (Figure 3 in Li *and others*, 2012), the proportion of false positives is not adequately low for all scenarios considered in the simulation study.

### 2.3. Proposed model

The proposed model follows the Poisson lognormal specification of the baseline model (Section 2.1), with Equations (2.1) to (2.4) remaining the same. We address the limitations of the baseline model in the rest of this section.

To make the approach able to deal with long time series, we allow the mixing parameter $z_i$ in Eq. 2.2 to become $z_{it}$; the probability of whether area $i$ is common or unusual therefore changes across time (Limitation 1). In addition, the posterior mean $z_{it}$ provides information on both where and when unusual behavior is observed (Limitation 2).

We allow the parameter $z_{it}$ to follow a hierarchical model:

(2.7) \begin{eqnarray*}z_{it} & \sim & \mbox{Bern}(\phi_{it}) \text{, where}\\\end{eqnarray*}

(2.8) \begin{eqnarray*}\mbox{logit}(\phi_{it}) & =& \pi_i + \delta_t + \mbox{logit}(\tau)\\\end{eqnarray*}

(2.9) \begin{eqnarray*}\tau &\sim& \mbox{U}(0.9, 1),\end{eqnarray*}

where $\mbox{logit}(\tau) = \log(\frac{\tau}{1-\tau}$). The spatial component $\pi_i$ is assigned an ICAR $\pi_i \sim \mbox{ICAR}(\textbf{W}, \sigma_\pi^2)$ and the temporal component $\delta_t$ is assigned the temporal analogue of ICAR, $\delta_t \sim \mbox{ICAR}(\textbf{Q}, \sigma_\delta^2)$, where $\textbf{W}$ and $\textbf{Q}$ are the spatial and temporal adjacency matrices respectively as specified in Section 2.1. Through this specification, we account for dependences of the unusual observations in space and time (Limitation 3); it is realistic to assume that the probability that area $i$ is unusual at time point $t$ depends on the corresponding probability of a neighboring area at time point $t$, and the corresponding probability of area $i$ at time points $t-1$ and $t+1$.

The hyperparameters $\sigma_\pi^2$ and $\sigma_\delta^2$ are assigned a weakly informative half Normal prior $N(0, 1)$. All remaining parameters and hyperparameters of Equations (2.2) to (2.4) are specified as in Section 2.1, except for the area-specific variance $\sigma_{\kappa_i}^2$ which now follows a less restrictive half Normal prior $N(0, 1)$. Given the hierarchical structure of $z_{it}$, and the fact that the proposed model is appropriate for longer time series, more information is now available, and therefore, it is no longer needed to specify an informative prior on the area-specific variances, but rather allow for more flexibility in the detection mechanism of the model.

We also include in the model the term $\mbox{logit}(\tau)$; this shifts the expectation of $\phi_{it}$ from 0.5 to parameter $\tau$ which is now assigned a prior distribution. The rationale behind the choice for the prior of $\tau$ is that it needs to be relatively high so that a small proportion of observations follows the *Area-Specific model* (roughly 5–10%), thus characterized as unusual, while at the same time it allows for flexibility to deal with scenarios with no unusual observations. We, therefore, specify a uniform prior with a range between 0.9 and 1, assuming that the proportion of unusual observations varies from 0 (when no unusual behavior is evident) to 10% (Limitation 4).

An additional positive impact of modeling $z_{it}$ hierarchically is that we achieve low proportions of false positives (Limitation 5), while we no longer need to incorporate the Bayesian FDR to adjust for the multiple testing problem. Within the Bayesian modeling framework, the interpretation of the posterior $z_{it}$ is straightforward; as long as the model is properly specified, accounting for known sources of variability, $f_{it}$ = P($z_{it}$=1$\mid$data) represents the posterior probability that area $i$ follows the common trend at time point $t$ (Gelman *and others*, 2012). We select areas with probability $f_{it}$ less than the standard statistical significance level of 0.05, and we class them as unusual at the corresponding time point/points $t$.

## 3. Simulation study

We carried out a simulation study to evaluate the performance of our proposed model under various realistic scenarios, as well as to compare it against the baseline model. For comparison purposes, we followed closely the simulation design used in Li *and others* (2012).

### 3.1. Data generation and study design

We generated count data for 211 areas in England over 15 time points under the *Common model* (Eq. 2.3). These areas represent Clinical Commissioning Groups, an authority responsible for decisions on health related matters at the local level. We used data on asthma hospital admissions in the same areas to (i) calculate the expected number of cases after adjusting for age and sex characteristics using indirect standardization and (ii) obtain posterior parameter estimates from the *Common model*, which were then used to simulate the outcome variable. Summary statistics of the asthma hospitalization data set can be found in Table 3 in the supplementary material available at *Biostatistics* online.

The simulated counts represent the behavior of the disease under normal conditions. In order to evaluate whether the model is able to pick up unusual behavior, we selected a number of areas to follow a temporal trend that differs from the national one. For the selection of areas, we considered two different spatial scenarios: (i) one where unusual areas are isolated (they do not share geographical borders) and (ii) one where they form clusters (they share geographical borders).

Accounting for the fact that the spatial risk and the magnitude of the expected cases will affect the ability of the model to detect a particular area, we selected 15 unusual areas in total (for details see Section 1 in the supplementary material available at *Biostatistics* online). For each of the two spatial scenarios, we considered three temporal scenarios: (i) one where unusual time points are isolated, (ii) one where they are consecutive and characterized by a high degree of variability, and (iii) one where they are consecutive and show a stable pattern, i.e. they do not show high variability (Figure 1a–c in the supplementary material available at *Biostatistics* online). This will allow us to evaluate whether the model is able to detect unusual behavior when spatial and temporal dependences are evident.

**Fig. 1.**
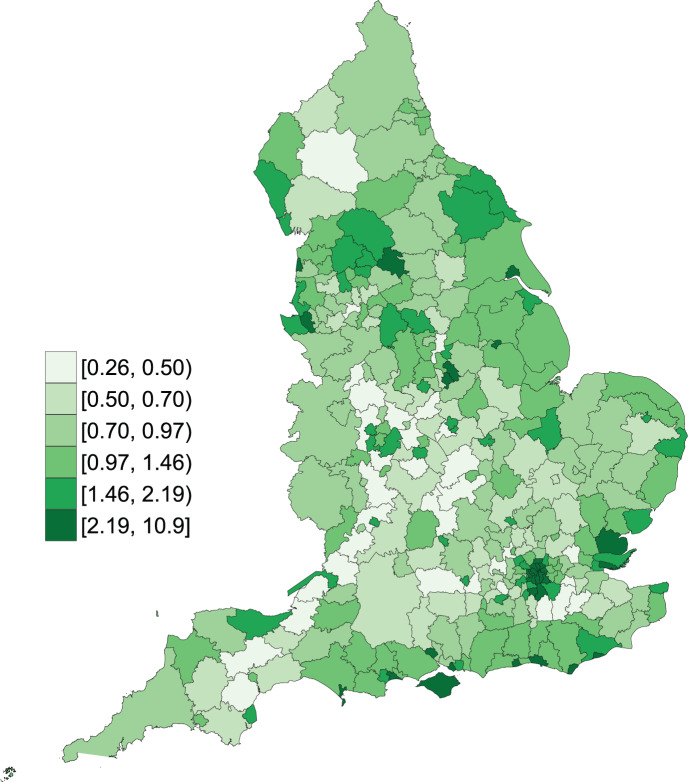
Map of posterior accident rates in England.

In addition to the above six scenarios, we also include the following: (i) one scenario with 30 time points in order to evaluate the performance of the model on much longer time series (Figure 1d in the supplementary material available at *Biostatistics* online), (ii) one scenario with reduced expected cases, and (iii) one scenario with increased expected cases, in order to assess the impact of the magnitude of the expected counts (see Table 3 in the supplementary material available at *Biostatistics* online). Finally, we consider (iv) one scenario with no aberrations in order to assess whether the model produces positives when there is no signal. All 10 scenarios are summarized in Table 1. Details about the construction of all unusual temporal trends can be found in Section 2 in the supplementary material available at *Biostatistics* online.

**Table 1.** Scenarios for the simulated data

Scenario	Spatial pattern	Temporal pattern	Expected cases
S1	Isolated	Isolated	Normal
S2	Isolated	Consecutive-variable	Normal
S3	Isolated	Consecutive-stable	Normal
S4	Clustered	Isolated	Normal
S5	Clustered	Consecutive variable	Normal
S6	Clustered	Consecutive stable	Normal
S7	Isolated	Long time series	Normal
S8	Isolated	Consecutive variable	Increased
S9	Isolated	Consecutive variable	Reduced
S10	No aberrations		

In order to ensure that the proposed model performs well also in the case of short time series, where the baseline model has shown to be appropriate, we carried out a simulation study with eight time points. This is presented in Section 3 in the supplementary material available at *Biostatistics* online.

Fifty simulated data were generated in total for each scenario.

### 3.2. Results

For scenarios $S1$–$S9$ (Table 1), we are interested to detect as many aberrations as possible while keeping a low proportion of false positives.

In the case of our proposed model, an aberration is a combination of area $i$ and time point $t$ for which $f_{it}$ is below 0.05. In the case of the baseline model, an aberration is an area $i$ for which $f_i$ is below threshold $C=f(k)$, which satisfies the rule described in Section 2.1 with the preset level $\alpha$ set to 0.05. Therefore, given the simulated data set of 15 time points and 211 areas, $\mbox{TN+TP+FP+FN} = 211*15$ for the proposed model, while $\mbox{TN+TP+FP+FN} = 211$ for the baseline model. Model performance was assessed through sensitivity $\big(\mbox{TP/(TP+FN)}\big)$, specificity $\big(\mbox{TN/(TN+FP})\big)$, proportion of false positives $\big(\mbox{FP/(TP+FP})\big)$, and global error $\big($(FP+FN)/(TN+TP+FP+FN)$\big)$, where TP, FP, TN, and FN are the numbers of true positives, false positives, true negatives, and false negatives, respectively. For the proportion of false positives, when there are no declared unusual observations ($\mbox{TP+FP} = 0$), this is set to 0. In order to assess the performance of the scenario with no aberrations ($S9$), we take the proportion of simulations (of the total 50) with at least one aberration declared, and the corresponding average number of these.

Table 2 presents the averaged values of the performance criteria across 50 simulations for scenarios $S1$ to $S9$. As can be seen, the mean proportion of false positives from the baseline model is above 0.2 for all scenarios, which says that at least 20% of the declared unusual observations are not truly unusual. The range of values is detailed by the scenario characteristics: the minimum, equal to 0.2, corresponds to the case where reduced expected cases are assumed, whereas the highest values, 0.32 and 0.33, correspond to the scenarios with a longer time series ($S7$) and increased expected cases ($S8$), respectively. From this we can conclude that when more information is added, i.e. the data are less sparse, the performance of the baseline model in terms of false positives becomes worse. Although sensitivity is very high for the same model, and specificity and global error have adequate values, the high proportion of false positives makes the model inappropriate to use for scenarios with long time series, i.e. 15 time points and above. In Table 1 in the supplementary material available at *Biostatistics* online, we can see that for short time series (eight time points), the baseline model is powerful and gives relatively low proportions of false positives, as has been previously shown.

**Table 2.** Performance for scenarios S1–S9; parentheses indicate 95% credible intervals (the 2.5 and 97.5 percentiles of the distribution from 50 simulations)

	FP proportion	Sensitivity	Specificity	Global error
Baseline model				
S1	0.250 (0.211, 0.286)	0.965 (0.933, 1.000)	0.976 (0.969, 0.980)	0.025 (0.019, 0.028)
S2	0.264 (0.211, 0.296)	1.000 (0.985, 1.000)	0.972 (0.969, 0.980)	0.027 (0.019, 0.032)
S3	0.271 (0.222, 0.318)	0.967 (0.933, 1.000)	0.972 (0.964, 0.980)	0.029 (0.024, 0.033)
S4	0.268 (0.250, 0.318)	1.000 (0.999, 1.000)	0.971 (0.964, 0.974)	0.027 (0.024, 0.033)
S5	0.286 (0.286, 0.318)	1.000 (1.000, 1.000)	0.969 (0.964, 0.969)	0.029 (0.028, 0.033)
S6	0.295 (0.250, 0.318)	1.000 (1.000, 1.000)	0.968 (0.964, 0.974)	0.030 (0.024, 0.033)
S7	0.322 (0.286, 0.306)	1.000 (0.933, 1.000)	0.963 (0.959, 0.969)	0.035 (0.028, 0.041)
S8	0.327 (0.286, 0.348)	1.000 (1.000, 1.000)	0.962 (0.959, 0.969)	0.035 (0.028, 0.038)
S9	0.200 (0.143, 0.222)	0.857 (0.800, 0.933)	0.984 (0.980, 0.990)	0.025 (0.019, 0.032)
Proposed model				
S1	0.022 (0.000, 0.036)	0.710 (0.671, 0.750)	1.000 (0.999, 1.000)	0.006 (0.005, 0.007)
S2	0.019 (0.015, 0.031)	0.796 (0.763, 0.827)	1.000 (0.999, 1.000)	0.005 (0.004, 0.006)
S3	0.150 (0.118, 0.187)	0.799 (0.762, 0.814)	0.998 (0.997, 0.999)	0.005 (0.004, 0.006)
S4	0.030 (0.000, 0.052)	0.881 (0.838, 0.917)	0.999 (0.999, 1.000)	0.003 (0.002, 0.003)
S5	0.029 (0.014, 0.041)	0.928 (0.907, 0.947)	0.999 (0.999, 1.000)	0.002 (0.002, 0.003)
S6	0.225 (0.190, 0.266)	0.932 (0.911, 0.956)	0.996 (0.995, 0.997)	0.005 (0.004, 0.006)
S7	0.027 (0.022, 0.034)	0.750 (0.710, 0.770)	0.973 (0.966, 0.978)	0.008 (0.007, 0.008)
S8	0.057 (0.040, 0.075)	0.969 (0.960, 0.987)	0.999 (0.998, 0.999)	0.002 (0.002, 0.003)
S9	0.012 (0.000, 0.027)	0.480 (0.427, 0.507)	1.000 (1.000, 1.000)	0.013 (0.012, 0.014)

Our proposed method, on the other hand, gives adequately high sensitivity values and low proportion of false positives for most scenarios (Table 2). More specifically, we observe that for scenarios $S1$, $S2$, $S4$, $S5$, $S7$, and $S8$, our proposed model outperforms the baseline model: sensitivity ranges from 0.71 to 0.97, whereas the proportion of false positives is always below 0.05. When the temporal pattern is stable (scenarios $S3$ and $S6$), the proportion of false positives increases to 0.15 and 0.23, respectively, still lower than the corresponding values of the baseline model, i.e. 0.26 and 0.29. In the case of reduced expected cases ($S9$), our model has a mean proportion of false positives equal to only 0.01, however, sensitivity is 0.5, indicating that it is not able to pick up unusual behavior sufficiently. In the case of short time series, it seems that the baseline model is slightly better than our proposed model overall, however, the associated mean proportion of false positives is higher than 0.05 for all scenarios.

In the extreme case of no aberrations ($S10$), a proportion of 8% simulations (of the total 50) produces positives, while these give an average number of declared aberrations equal to 1.25, contrary to a corresponding 26% with an average 1.31 produced by the baseline model (Table 3). This suggests that the proposed model seldom produces false positives, and when this happens, the corresponding number tends to be very small.

**Table 3.** Performance for scenario S10

	Baseline model	Proposed model
Simulations that detect at least one aberration (%)	26	8
Mean number of aberrations detected	1.31	1.25

In order to show that the model parameters are estimated well, the simulated temporal trend under the *Common model* is plotted against the corresponding estimated trends for scenarios $S1$–$S6$ (Figure 3 in the supplementary material available at *Biostatistics* online). In addition, the simulated temporal trend under the *Area-Specific model* is plotted against the estimated trends of the 15 unusual areas for scenarios $S1$–$S6$ (Figure 4 in the supplementary material available at *Biostatistics* online).

## 4. Case study: road traffic accidents in England

We applied the proposed method to a set of road traffic accidents data at small area level in England over a period of 11 years. Road traffic accidents are considered to be a major public health issue, and they are typically analyzed by methods developed for non-communicable diseases (Miaou *and others*, 2003; MacNab, 2003; Song *and others*, 2006; Aguero-Valverde and Jovanis, 2008; Boulieri *and others*, 2017). We seek to describe the overall risk across space and time, while we focus on the detection of areas with unusual temporal trends.

### 4.1. Data

Road traffic accidents data in Great Britain are recorded on a STATS19 report from police officers and are held by the Department for Transport. The data include information on the postcode, date, and severity level (slight, severe, or fatal) of each accident. We analyze severe and fatal accidents data together for 326 districts in England over the years 2005–2015.

The annual average daily flow (AADF) data for the major road network (motorways and A-roads) in England were obtained from the Department for Transport. These are the traffic counts for each junction-to-junction link on the major roads containing spatial information in the form of a postcode. The AADF data set was joined to the major road network shapefile, also obtained from the Department for Transport. Each AADF point was associated to a road segment based on unique road names, or, if not available, based on distance. For the road segments with no corresponding AADF count, this was estimated as the average AADF of the bordering road segments, and in the few occasions when this was not possible the middle value of AADF was used.

The resulting shapefile, containing information on all road segments in England with their corresponding AADF values was intersected with the districts shapefile. The traffic volume of each intersected road segment was then calculated by multiplying the length of the road segment by its AADF value. Finally, the district level traffic volume was estimated by summing the traffic volumes of all road segments lying within each district:

(4.1) \begin{eqnarray*}\mbox{TV}_{\text{D}} &=& \sum_{\text{rs} \in \text{D}}{\text{TV}_\text{rs}} \\\end{eqnarray*}

(4.2) \begin{eqnarray*}\mbox{TV}_{\text{rs}} &=& \text{length(rs)} \; \text{AADF}_{\text{rs}}\end{eqnarray*}

where TV is the traffic volume, D represents the district level and rs represents the road segment.

The traffic volume was calculated for all years considered in the study, i.e. 2005–2015, and the correlation was found to be 0.99. We, therefore, decided to use the traffic volume for the middle year 2010 for our analysis. The summary statistics for the accidents data and the accident rates (number of accidents divided by traffic volume) are presented in Tables 4 and 5 in the supplementary material available at *Biostatistics* online.

### 4.2. Results

The model was implemented in R using the R2OpenBUGS package. We run two chains for 80 000 MCMC iterations storing every 2nd iteration, from which 20 000 were discarded as a burn-in. The model took roughly 5 h on an Intel Core processor at 3.40 GHz with 16 Gbytes of RAM. We carried out convergence diagnostics through visual check of the trace plots, Brooks–Gelman–Rubin statistic, autocorrelation plots, and assessing the Monte Carlo error (see Figure 5 in the supplementary material available at *Biostatistics* online).

**Fig. 2.**
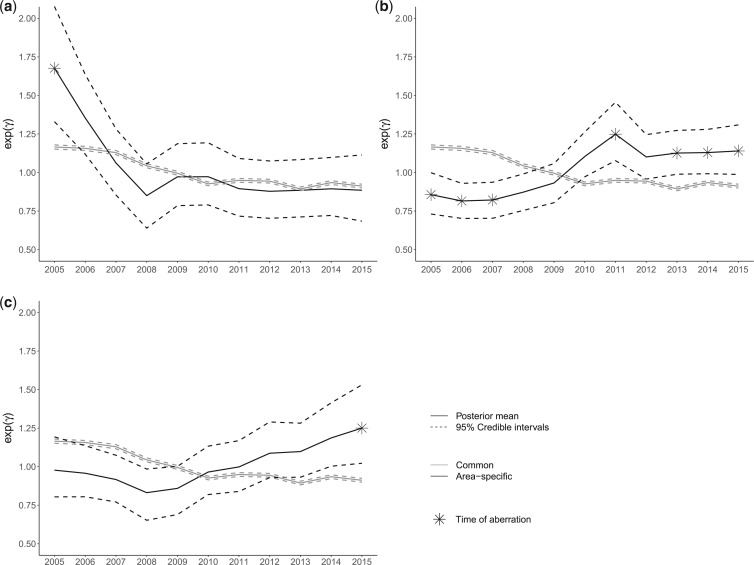
The national trend of England against the time trends of three selected unusual areas. (a) District of Eden. (b) District of Southampton. (c) District of Elmbridge.

Figure 1 plots the posterior estimate of the spatial component $\eta_i$ (Eq. 2.3) on the exponential scale showing the overall pattern of accident risk across England; in the disease mapping context this is usually referred to as a relative risk, however, in our example, where the offset is the traffic volume and not an expected count of accidents, we interpret it as an accident risk. The map suggests higher accident risk in London and other big cities such as Manchester and Leeds, while low accident risk is observed along certain motorways such as M1 and M5. The posterior estimate of the temporal component $\gamma_t$ (Eq. 2.3) on the exponential scale, shown in Figure 2, shows the pattern of accident risk over the period 2005–2015, which can be thought of as the national trend. There is a nearly linear downward trend over time up until 2010, while afterwards the trend becomes flatter.

Under the model, 96 observations were detected as unusual out of which 45 correspond to unique areas (see map in Figure 6 in the supplementary material available at *Biostatistics* online). These are in the form of isolated areas as well as clusters. We present the area-specific temporal trends of three selected areas plotted against the national trend in Figure 2. These correspond to the parameter $\kappa_{i_t}$ (Eq. 2.4) on the exponential scale, where $i$ is the selected unusual area.

The district of Eden in Cumbria shows a temporal trend with a much higher accident rate than the national average at the beginning of the time period, with an aberration detected by the model in year 2005. According to published reports, the rates of deaths and serious injuries in Cumbria are significantly higher than the national average, with the district of Eden having the highest rate in the county (Brown, 2015; Critchley and Whitfield, 2015).

The district of Southampton follows a nearly opposite temporal pattern to the national one, with aberrations detected at several time points over the study period and the highest accident risk observed in 2011. This might be related to the increased popularity of cycling in Southampton potentially leading to high accident rates. In fact, the time trend of the accident risk in Southampton somewhat resembles the trend corresponding to cyclist numbers in the area (Southampton City Council, 2016).

Similarly, the district of Elmbridge in Surrey exhibits an overall upward trend with an aberration detected in year 2015, a finding which is in line with recent reports ranking Surrey among the areas with the sharpest increase in the numbers of people killed or seriously injured on the roads (Surrey Police, 2015). In specific, road accidents involving vans and lorries increased by a third in Surrey in 2015 compared to the previous year, while the number of pedestrians who were injured on the roads of Elmbridge rose by 54% for the same year (Scott, 2016a, 2016b).

## 5. Discussion

In this article, we have described a Bayesian spatio-temporal model for public health surveillance that can be used for detection of areas with unusual behavior over time. We use the BaySTDetect method as a baseline, and we overcome its limitations by extending it to a more generic framework. Allowing the mixing parameter to change across time results in a model which is appropriate for long time series, a case where the baseline model showed lack of performance. Information on the time points when unusual behavior occurs is also provided to facilitate interpretation and etiological investigation. The prior of the mixing parameter changes to a less informative hierarchical prior that accounts for spatial and temporal dependences making the model more flexible for detection of several realistic unusual patterns.

Surveillance methods are typically optimized for one sole criterion depending on the problem in hand. In epidemiological surveillance, where the financial resources are usually limited and the priority is to spend expenditure wisely, we need to stress the importance of controlling the proportion of false positives. The baseline model, for instance, which is able to correctly detect nearly all unusual observations as such, at the cost of excess numbers of false detections, is not appropriate in this context. Our proposed model, on the other hand, has the great advantage of keeping the proportion of false positives below 5% for most scenarios. This suggests that we can be confident that the observations that are detected as unusual under the model are indeed unusual. In addition, unusual behavior is detected only when this is evident, as shown from the scenario with no aberrations ($S10$), thus confirming our hypothesis that the strong prior specification on $z_i$ of the baseline model (Eq. 2.8) induces bias when this does not agree with the data. At the same time, the corresponding sensitivity and specificity values are satisfactory.

An additional strength of the proposed model is that there is no need to adjust for the multiple testing problem as in the case of the baseline model. This arises when multiple tests are performed, and false detections are likely to occur due to pure chance. Although the importance of controlling for multiple testing is clear in classical inference, the relevant problem in the Bayesian setting is not always considered valid. Gelman *and others* (2012) argue that when the data are properly modelled through a Bayesian hierarchical framework, there should be no concern about false detections; the estimates are naturally smoothed towards the mean and they become more conservative and therefore less prone to false positives. On the other hand, it is likely that the estimates suffer from a higher proportion of false negatives which may lead to under-detection of important risks.

We note that the model is designed specifically for detection of a small proportion of unusual observations when these exist, ranging a priori between 0 and 10%. The upper bound could be chosen based on each specific case; however, a much larger proportion of unusual trends, say 20% and more, would not be seen as unusual but rather as a different common trend. The objective would then be clustering of multiple time trends, and a model following non-parametric Bayesian approaches would be more appropriate (see for instance Dass *and others*, 2015).

From a policy making perspective, the proposed model could be especially valuable for healthcare professionals who can use it for spatial monitoring at the population level, in order to detect areas with unusual behavior over time. This might indicate the impact of a policy/intervention, or the presence of a localized risk factor. Detecting areas with increased demand is particularly important for implementation of targeted intervention strategies, however, further investigation would be normally required to better understand unusual patterns, and generate hypotheses regarding associated risk factors.

While our model has shown its potential in detecting unusual patterns under various temporal and spatial scenarios, its performance was less satisfactory when the unusual time trend was stable (i.e. consecutive-stable patterns), as it gave a relatively high proportion of false positives. On the other hand, when the unusual trend was more variable (i.e. isolated and consecutive-variable patterns), the proportion of false positives was very low, at the cost of a slight decrease in power. In order to test whether this is due to the space-time separable structure of the probability model in Eq. 2.10, we carried out two simulations using a modified version of the model, where an additional latent variable is included to allow for space-time interactions. The results show that the additional parameter does not provide any important benefit on the performance of the model, suggesting that the conservative performance of our model is not due to the lack of flexibility on the parameter $\phi_{it}$ in Eq. (2.10). Instead, it is more related to the overall hierarchical structure of the parameter $z_{it}$, which being more flexible, it is more appropriate for longer time series, while addressing the issues of identifiability and multiple testing. This results in a low proportion of false positives, which comes at the cost of losing slightly in power. Details about the additional simulations and the corresponding results can be found in Section 4 in the supplementary material available at *Biostatistics* online.

In addition, the model was not able to adequately pick up signal when expected cases were reduced to half. This might be due to the area-specific variances, which are defined on a specific scale of data. Potentially a way to address this is to assume that these are relative to the variance of the *Common model*.

It was also shown that for short time series (eight time points) there is no additional benefit of using our proposed model rather than the baseline model, suggesting that it is probably too complex for such short time series. We, therefore, recommend that the baseline model should be used for times series of length less than 10, as was also recommended by Li *and others* (2012), while the proposed model should be preferred otherwise. In the context of epidemiological surveillance, nevertheless, the availability of data has recently shifted the public interest from yearly to monthly or weekly time resolution, hence resulting in longer time series where the proposed approach would be beneficial.

An example of such analyses is the syndromic surveillance which makes use of data on pre-diagnostic syndromes rather than confirmed cases of specific diseases, such as ambulance calls or drug prescriptions, with the aim to provide early warning detection (Zou *and others*, 2011; Corberán-Vallet and Lawson, 2014). A natural extension of the proposed model would be the prospective setting, such that syndromic surveillance can be performed.

The mixture modeling approach that we propose is specifically designed for detection of unusual behavior allowing the implementation of two separate components. Alternatively, it is common in the disease mapping context to assume a single component model where an interaction term is used to highlight spatio-temporal deviations. A comparison among the two approaches through a simulation study would be useful to identify their respective benefits and drawbacks.

Furthermore, we are currently developing a web application that integrates the proposed model and allows for easy implementation and the creation of interactive data visualizations. This could be particularly useful to public health researchers and practitioners who may lack adequate programming skills and advanced statistical knowledge to carry out complex analyses.

## 6. Software

The code is provided on Github at https://github.com/aretib/bayes_mixture.git.

## Supplementary Material

kxy038_Supplementary_Materials
