# Supplementary material for: A Bayesian mixture modeling approach for public health surveillance
Source: Biostatistics. 2018 Sep 25;21(3):369–83. doi: 10.1093/biostatistics/kxy038 (PMC7307974; doi:10.1093/biostatistics/kxy038)
Supplement: kxy038_Supplementary_Materials [file kxy038_supplementary_materials.pdf]

Biostatistics (2018), **0**, 0, *pp.* 1–11
doi:10.1093/biostatistics/Submission3˙supplementary˙materials

A Bayesian mixture modeling approach for public health surveillance 'Supplementary Materials'

ARETI BOULIERI*, JAMES E BENNETT, MARTA BLANGIARDO

MRC- PHE Environment and Health, Department of Epidemiology and Biostatistics, Imperial College London, Norfolk Place, W2 1PG, London, UK

a.boulieri@imperial.ac.uk

1. Selection of 15 unusual areas

Unusual areas were selected under two different spatial scenarios: i) isolated and ii) clustered. In order to obtain a good representation of areas, these were selected based on a range of expected cases and spatial risk η_i as we expect that the levels of these will affect the ability of the model to detect a particular area.

For scenario i, we selected 3 areas from each of the percentiles 10th, 25th, 50th, 75th and 90th. At each percentile, each of the 3 areas corresponds to one of the 3 levels of the overall spatial risk η_i: low (within the 10th-30th percentiles), medium (within the 45th-55th percentiles) and high (within the 70th-90th percentiles). For spatial scenario ii, we selected 3 clusters consisting of 4, 5, and 6 areas respectively. For each of these clusters, areas with a variety of levels in their expected cases and spatial risk were selected. Under each scenario, 15 areas were selected to be

*To whom correspondence should be addressed.

© The Author 2018. Published by Oxford University Press. All rights reserved. For permissions, please e-mail: journals.permissions@oup.com

unusual.

2. Construction of unusual temporal patterns

If γ_t, for $t = 1, \ldots, 15$, is the common time trend, and γ_t^* is the time trend corresponding to the unusual areas, then we assumed the following 4 temporal scenarios:

1. Isolated: $\gamma_t^* = \gamma_t + \log(2)$, for t = {3, 10}, $\gamma_t^* = \gamma_t - \log(2)$, for t = {6, 12, 15}, and $\gamma_t^* = \gamma_t$ otherwise.

2. Consecutive-variable: $\gamma_t^* = \gamma_t - \log(2)$ for t = 11, $\gamma_t^* = \gamma_t - \log(2.2)$ for t = {12, 15}, $\gamma_t^* = \gamma_t + \log(2.2)$ for t = 13, $\gamma_t^* = \gamma_t + \log(1.8)$ for t = 14, and $\gamma_t^* = \gamma_t$ otherwise.

3. Consecutive-stable: $\gamma_t^* = \gamma_t + \log(2)$, for t = 1, $\gamma_t^* = \gamma_{t-1} + \epsilon$, for t = {2, 3}, where ϵ represents small random noise, and $\gamma_t^* = \gamma_t$ otherwise.

4. Longer time series: $\gamma_t^* = \gamma_t + \log(2)$, for t = {6, 14, 25, 30}, $\gamma_t^* = \gamma_t - \log(2)$, for t = {11, 23} and $\gamma_t^* = \gamma_t + \log(2.2)$, for t = 13, $\gamma_t^* = \gamma_t - \log(2.2)$, for t = {12, 15} and $\gamma_t^* = \gamma_t$ otherwise.

Figure 1 shows the unusual temporal patterns corresponding to the above scenarios.

3. Simulation study: Short time series

We carried out a simulation study where 8 time points are considered, following closely the simulation design of the original paper by Li and others (2012). The spatial scenario with isolated unusual areas was used.

If γ_t, for $t = 1, \ldots, 8$, is the common time trend and γ_t^* is the time trend corresponding to the unusual areas, we assumed the following 3 temporal scenarios:

1. Scenario 1: $\gamma_t^* = \gamma_t + \log(2)$ for t = {1, 7}, $\gamma_t^* = \gamma_t - \log(2)$ for t = 4, and $\gamma_t^* = \gamma_t$ otherwise.

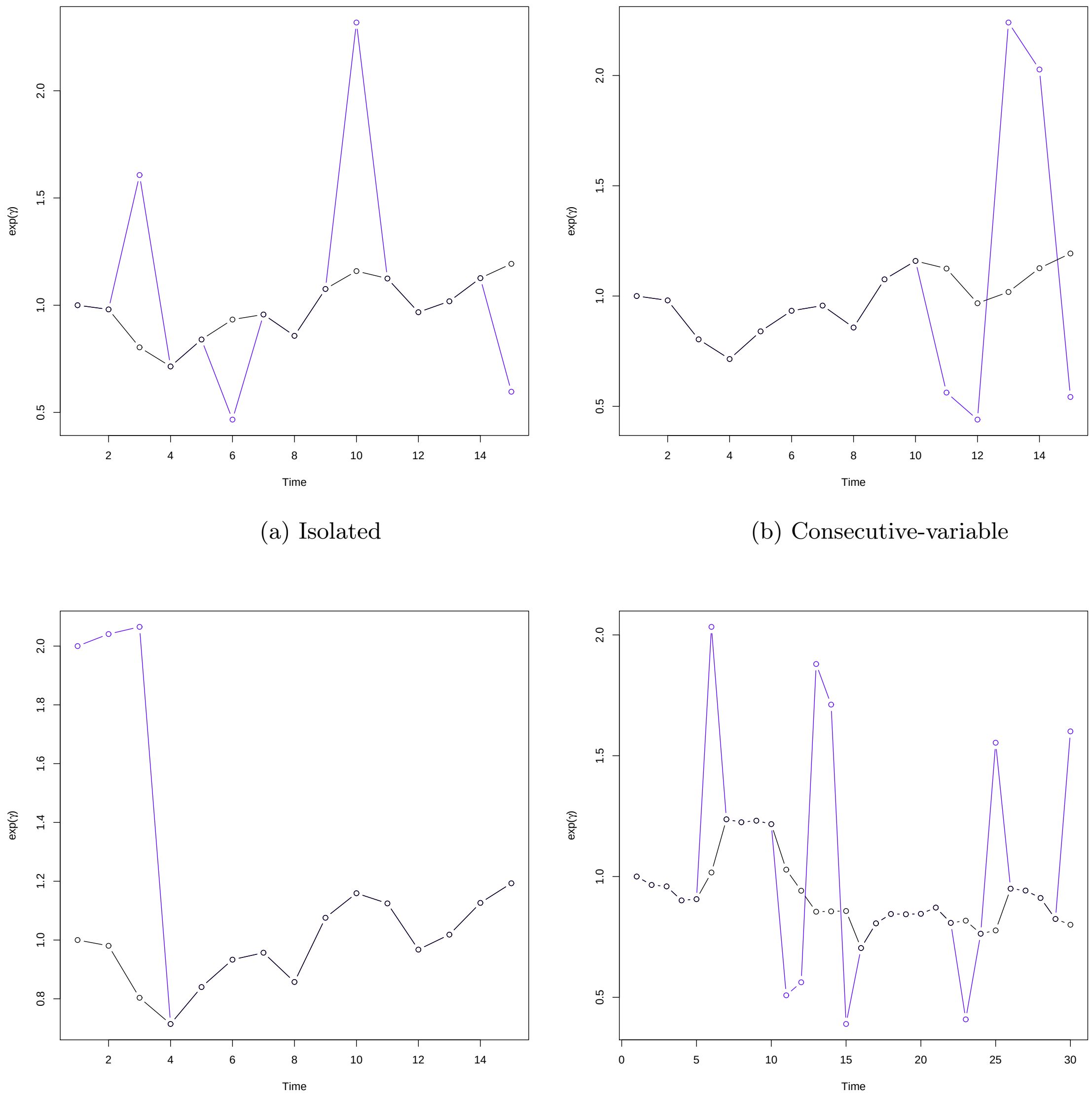

(a) Isolated

(b) Consecutive-variable

(c) Consecutive-stable

(d) Longer time series

Fig. 1: Temporal scenarios for the main simulation study (15 and 30 time points)

2. Scenario 2: $\gamma_t^* = \gamma_t + \log(2)$ for t = {3, 4}, and $\gamma_t^* = \gamma_t$ otherwise.

3. Scenario 3: $\gamma_t^* = \gamma_t + \log(2)$ for t = {1, 2}, and $\gamma_t^* = \gamma_t$ otherwise.

Figure 2 shows the unusual temporal patterns plotted against the common one. Results from

the simulation study with short time series can be seen in Table 1.

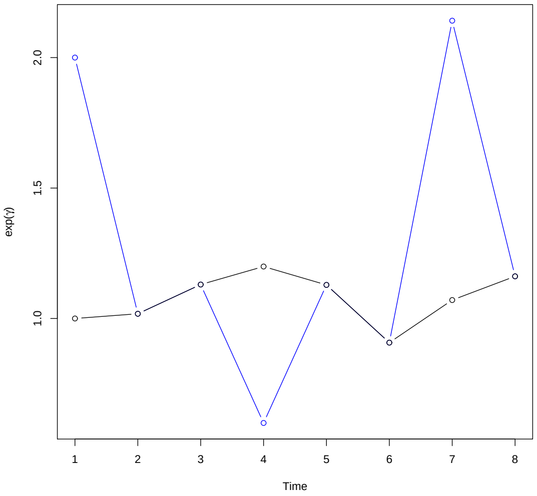

(a) Pattern 1

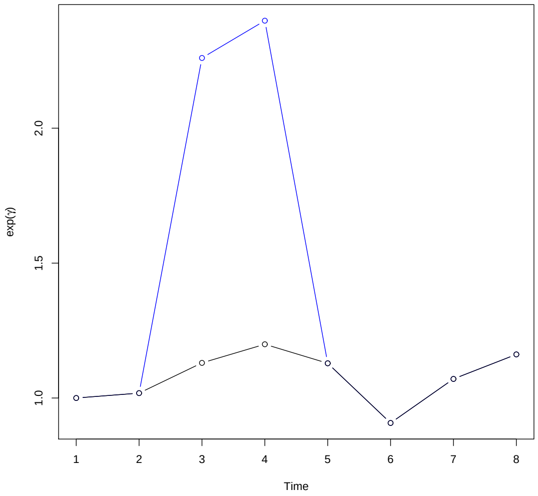

(b) Pattern 2

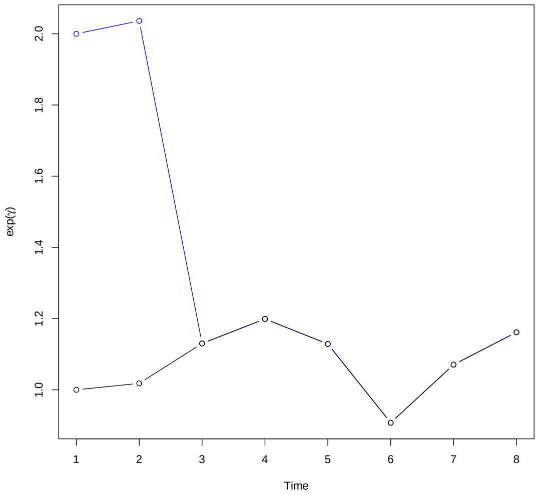

(c) Pattern 3

Fig. 2: Temporal scenarios for the simulation study with short time series (8 time points)

Table 1. *Performance for the simulation study with short time series; 95 %CI are in parentheses.*

	%FP	sensitivity	specificity	global error
Baseline model				
Scenario 1	0.079(0.062,0.125)	0.885(0.867,0.933)	0.994(0.990,0.995)	0.014(0.009,0.014)
Scenario 2	0.089(0.064,0.133)	0.889(0.867,0.933)	0.993(0.990,0.995)	0.014(0.009,0.019)
Scenario 3	0.059(0.00,0.083)	0.844(0.750,0.933)	0.996(0.995,1.00)	0.015(0.009,0.019)
Proposed model				
Scenario 1	0.030(0.025,0.054)	0.733(0.689,0.778)	0.996(0.999,0.999)	0.011(0.007,0.008)
Scenario 2	0.159(0.121,0.192)	0.667(0.600,0.733)	0.998(0.997,0.998)	0.008(0.007,0.009)
Scenario 3	0.080(0.046,0.115)	0.715(0.642,767)	0.999(0.998,0.999)	0.006(0.006,0.007)

4. Simulation study: Modified model

We carried out two additional simulations using a modified version of the proposed model. Eq. 2.7 is as follows:

$$\log(\phi_{it}) = \pi_i + \delta_t + h_{it} + \text{logit}(\tau), \tag{4.1}$$

where $h_{it} \sim N(0, \sigma^2)$, with σ^2 following a weakly informative half Normal prior. The scenarios that we selected for the simulations were S1 and S6; S1 corresponds to the 'isolated time pattern and isolated areas' and gave a very good performance under our proposed model, while S6 corresponds to the 'consecutive-stable time pattern and clustered areas' and gave poor performance respectively (see Table 2 in main paper).

Comparison between the model above (Modified model) and our model (Proposed model) for scenarios S1 and S6 is presented in Table 2. The proportion of false positives increases from 0.022 to 0.044 for S1, and from 0.225 to 0.273 for S6, while sensitivity also increases, but less importantly. This suggests that although there is some gain in the power of the model, the loss in terms of false positives is more substantial, which might be due to overparameterisation of the model.

Table 2. *Comparison of performance between the proposed and the modified model for scenarios S1 and S6; 95 %CI are in parentheses.*

	%FP	sensitivity	specificity	global error
Proposed model				
S1	0.022(0.000,0.036)	0.710(0.671,0.750)	1.000(0.999,1.000)	0.006(0.005,0.007)
S6	0.225(0.190,0.266)	0.932(0.911,0.956)	0.996(0.995,0.997)	0.005(0.004,0.006)
Modified model				
S1	0.044 (0.021, 0.062)	0.725 (0.683, 0.763)	0.999(0.999,1.000)	0.006(0.005,0.006)
S6	0.273(0.242,0.311)	0.942(0.911,0.972)	0.995(0.994,0.996)	0.006(0.005,0.007)

5. Additional material

Table 3. *Summary statistics for expected data used for the main simulation study (asthma hospitalisation counts)*

	Min	1st Quartile	Median	Mean	3rd Quartile	Max
Normal	8	22	29	33.2	39	108
Increased	16	44	58	66.41	78	216
Reduced	4	11	14.5	16.6	19.5	54

Table 4. *Summary statistics for raw accidents data at district level*

Year	Q2.5%	Mean	Q97.5%
2005	44	74.8	89.8
2006	44	74.2	89
2007	43	72.5	87
2008	40	66.9	79
2009	38.3	63.9	75.8
2010	34.3	59.3	72.8
2011	34	60.9	72.8
2012	35.3	60.6	70
2013	34.3	57.2	66.8
2014	37	60	70
2015	36	58.5	69

Table 5. *Summary statistics for accident rates data at district level*

Year	Q2.5%	Mean	Q97.5%
2005	0.025	0.045	0.055
2006	0.024	0.046	0.053
2007	0.024	0.044	0.051
2008	0.022	0.041	0.050
2009	0.021	0.040	0.047
2010	0.019	0.036	0.042
2011	0.019	0.037	0.043
2012	0.020	0.037	0.042
2013	0.018	0.035	0.041
2014	0.020	0.038	0.047
2015	0.020	0.036	0.043

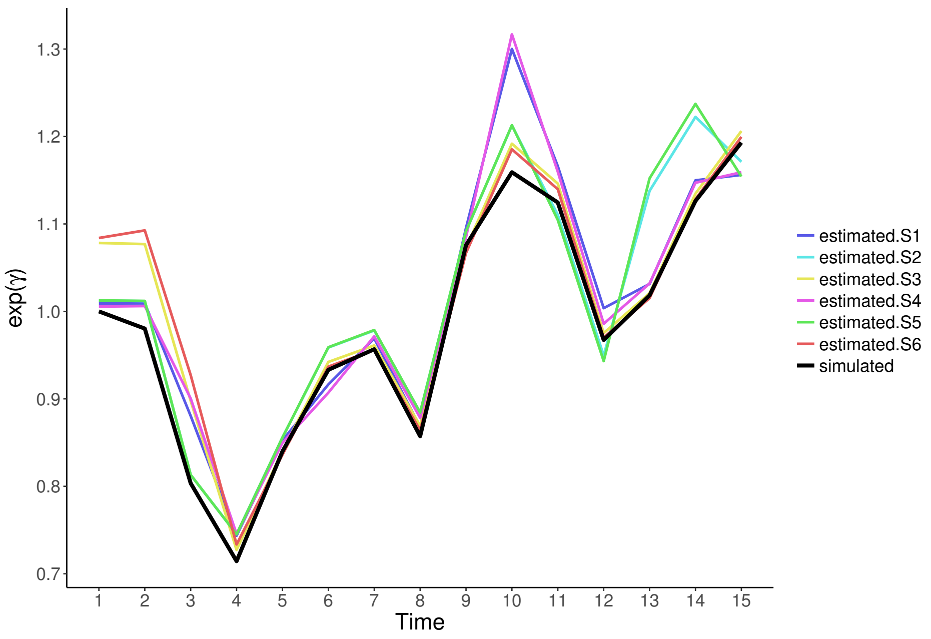

Fig. 3: Simulated common trend plotted against estimated posterior means for scenarios S1 to S6

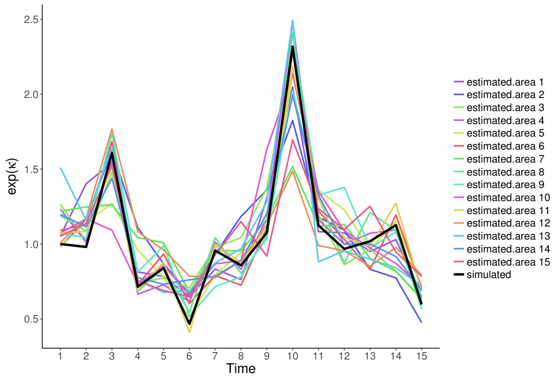

(a) S1

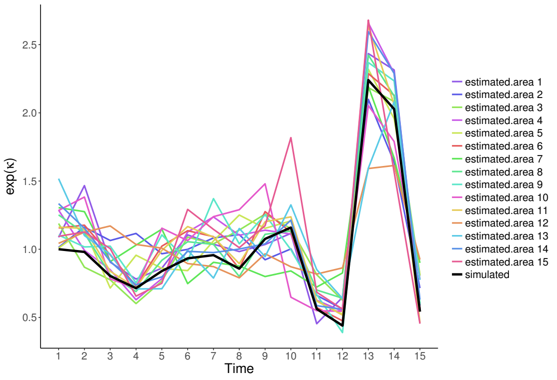

(b) S2

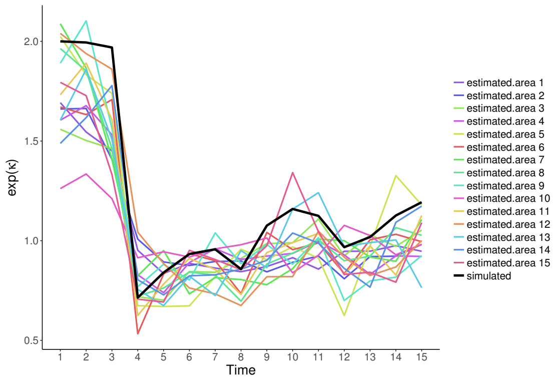

(c) S3

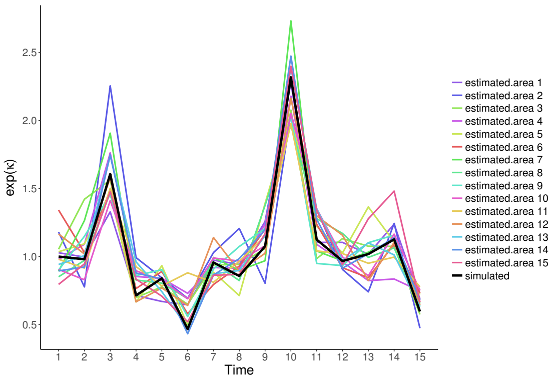

(d) S4

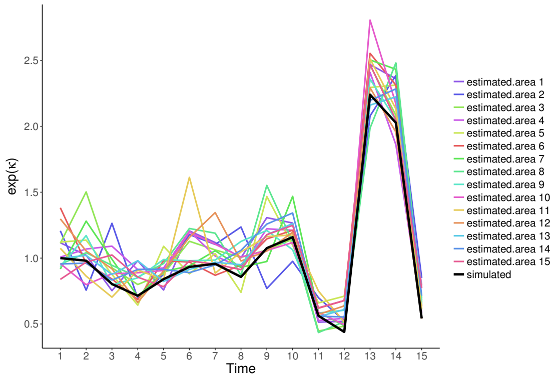

(e) S5

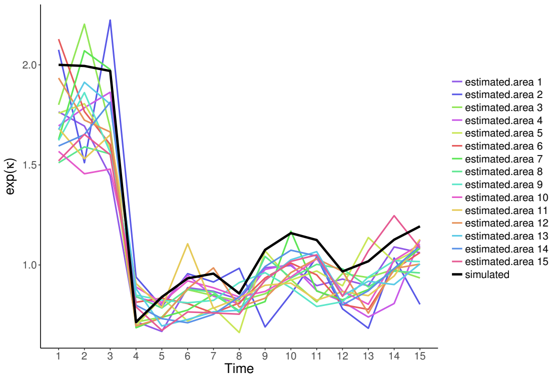

(f) S6

Fig. 4: Simulated unusual trend plotted against estimated posterior means for 15 unusual areas for scenarios S1 to S6

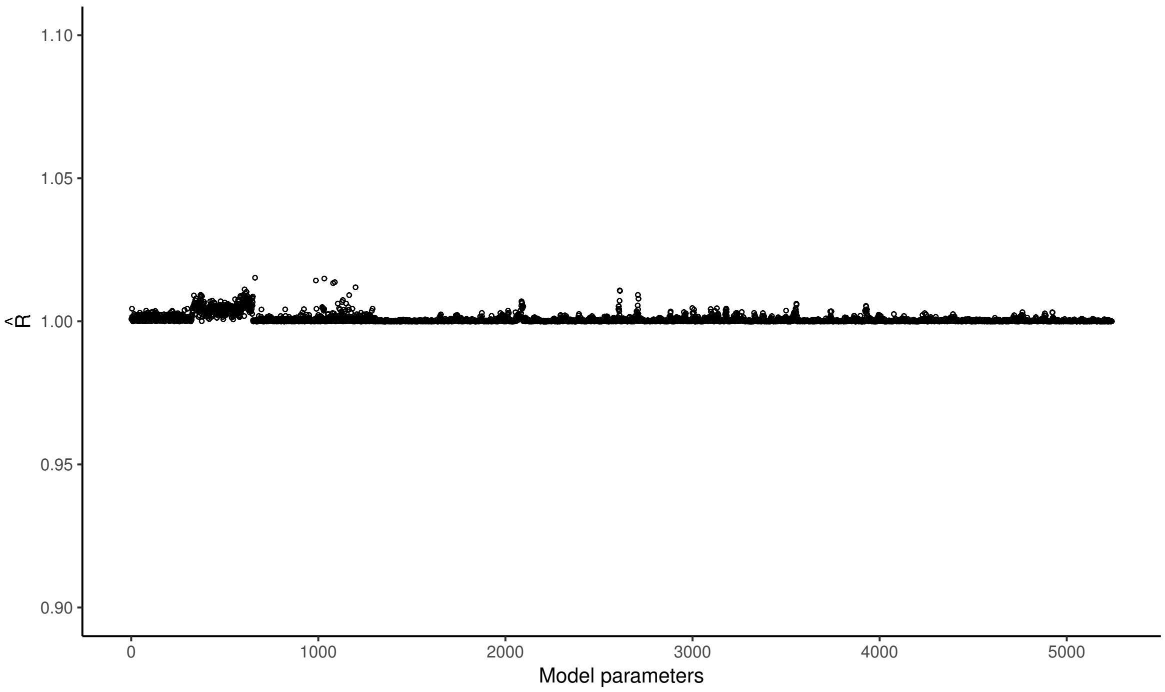

Fig. 5: Degree of convergence $\hat{R}$ estimated by the Brooks-Gelman-Rubin statistic across all model parameters (case study on road traffic accidents data)

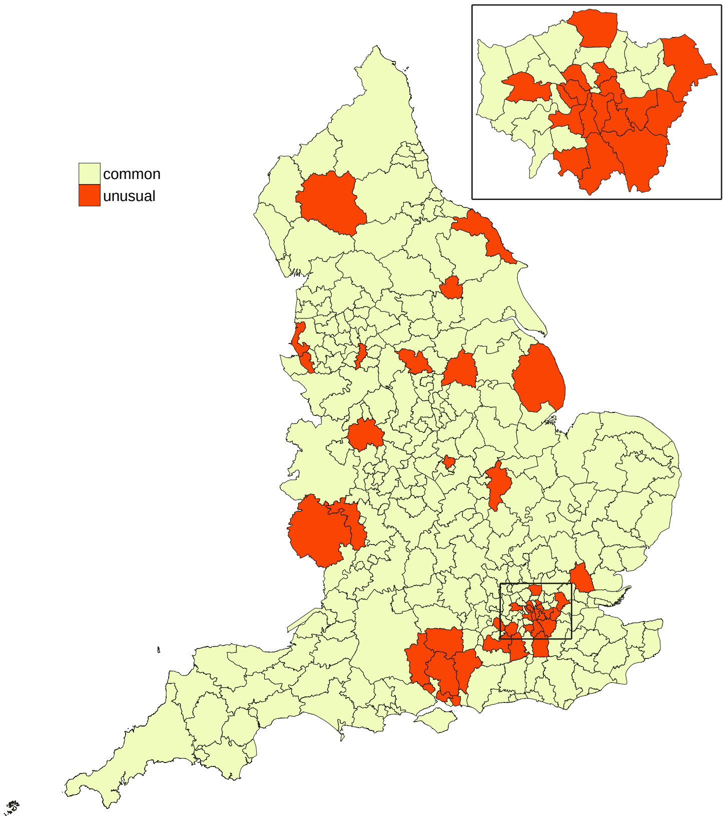

Fig. 6: Map of areas with common and unusual accident risk in England with London districts enlarged (case study on road traffic accidents data)
